# Evaluation of the Association between Persistent Organic Pollutants (POPs) and Diabetes in Epidemiological Studies: A National Toxicology Program Workshop Review

**DOI:** 10.1289/ehp.1205502

**Published:** 2013-05-07

**Authors:** Kyla W. Taylor, Raymond F. Novak, Henry A. Anderson, Linda S. Birnbaum, Chad Blystone, Michael DeVito, David Jacobs, Josef Köhrle, Duk-Hee Lee, Lars Rylander, Anna Rignell-Hydbom, Rogelio Tornero-Velez, Mary E. Turyk, Abee L. Boyles, Kristina A. Thayer, Lars Lind

**Affiliations:** 1 Office of Health Assessment and Translation, Division of the National Toxicology Program, National Institute of Environmental Health Sciences, National Institutes of Health, Department of Health and Human Services, Research Triangle Park, North Carolina, USA; 2 Shriners Hospitals for Children International, Tampa, Florida, USA; 3 Wisconsin Division of Public Health, Bureau of Environmental Health, Madison, Wisconsin, USA; 4 National Institute of Environmental Health Sciences, National Institutes of Health, Department of Health and Human Services, Research Triangle Park, North Carolina, USA; 5 Toxicology Branch, Division of National Toxicology Program, National Institute of Environmental Health Sciences, National Institutes of Health, Department of Health and Human Services, Research Triangle Park, North Carolina, USA; 6 Division of Epidemiology & Community Health, University of Minnesota School of Public Health, Minneapolis, Minnesota, USA; 7 Institute of Experimental Endocrinology, Charité Universitätsmedizin, Humboldt University, Berlin, Germany; 8 Department of Preventative Medicine, School of Medicine, Kyungpook National University, Daegu, Republic of Korea; 9 Division of Occupational and Environmental Medicine, Lund University, Lund, Sweden; 10 National Exposure Research Laboratory, U.S. Environmental Protection Agency, Research Triangle Park, North Carolina, USA; 11 Division of Epidemiology and Biostatistics, School of Public Health, University of Illinois-Chicago, Chicago, Illinois, USA; 12 Department of Medical Sciences, Uppsala University, Uppsala, Sweden

**Keywords:** chemically induced, diabetes, environment, epidemiology, glucose, hormone, insulin, metabolic syndrome, obesity, persistent organic pollutants, pollution, toxicology

## Abstract

Background: Diabetes is a major threat to public health in the United States and worldwide. Understanding the role of environmental chemicals in the development or progression of diabetes is an emerging issue in environmental health.

Objective: We assessed the epidemiologic literature for evidence of associations between persistent organic pollutants (POPs) and type 2 diabetes.

Methods: Using a PubMed search and reference lists from relevant studies or review articles, we identified 72 epidemiological studies that investigated associations of persistent organic pollutants (POPs) with diabetes. We evaluated these studies for consistency, strengths and weaknesses of study design (including power and statistical methods), clinical diagnosis, exposure assessment, study population characteristics, and identification of data gaps and areas for future research.

Conclusions: Heterogeneity of the studies precluded conducting a meta-analysis, but the overall evidence is sufficient for a positive association of some organochlorine POPs with type 2 diabetes. Collectively, these data are not sufficient to establish causality. Initial data mining revealed that the strongest positive correlation of diabetes with POPs occurred with organochlorine compounds, such as *trans*-nonachlor, dichlorodiphenyldichloroethylene (DDE), polychlorinated biphenyls (PCBs), and dioxins and dioxin-like chemicals. There is less indication of an association between other nonorganochlorine POPs, such as perfluoroalkyl acids and brominated compounds, and type 2 diabetes. Experimental data are needed to confirm the causality of these POPs, which will shed new light on the pathogenesis of diabetes. This new information should be considered by governmental bodies involved in the regulation of environmental contaminants.

Diabetes is a major threat to public health in the United States and worldwide [Centers for Disease Control and Prevention (CDC) 2011; Danaei et al. 2011; World Health Organization (WHO) 2011]. Whereas type 1 diabetes (T1D) is largely thought to be of an autoimmune origin, type 2 diabetes (T2D) is mainly associated with obesity and metabolic syndrome, although T2D can occur independently of overweight or obesity. Based on data from the 2005–2008 National Health and Nutrition Examination Survey (NHANES), 25.6 million, or 11.3%, of all people in the United States ≥ 20 years of age are estimated to have diagnosed or undiagnosed diabetes, with associated direct medical costs and indirect costs (disability, work loss, premature death) of $174 billion in 2007 alone (CDC 2011). Another 35% of people ≥ 20 years of age are believed to be prediabetic, a condition in which fasting blood glucose, blood glucose following a 2-hr oral glucose tolerance test (OGTT), or plasma HbA1c levels are above normal but not sufficiently elevated to be classified as diabetes (CDC 2011). The prediabetic condition often portends the subsequent development of T2D and is a risk factor for micro- and macrovascular diseases (Tabák et al. 2012).

Approximately 11% of prediabetic patients who participated in the Diabetes Prevention Program, a large multicenter randomized clinical trial developed by the National Institute of Diabetes and Digestive and Kidney Diseases (NIDDK), developed T2D each year during the average 3 years of follow-up (American Diabetes Association 2011; Knowler et al. 2002). Recently, T2D is being diagnosed in individuals earlier in life, including adolescents (NIDDK 2011). Given the number of people impacted by the disease, an estimated 346 million people worldwide (WHO 2011), and the long-term consequences of diabetes in terms of morbidity, mortality, and economic costs, there is considerable interest in understanding the contribution of “nontraditional” risk factors, such as environmental chemicals, to the diabetes epidemic. Environmental exposures that have been linked to diabetes in at least some study populations include persistent organic pollutants (POPs), arsenic, bisphenol A, phthlatates, organotins, nonpersistent pesticides (Thayer et al. 2012), and air pollution (Coogan et al. 2012; Hathout et al. 2006; Krämer et al. 2010; O’Neill et al. 2007; Pearson et al. 2010).

Over the past several years, research addressing the role of environmental chemicals in T2D has rapidly expanded. The February 2011 Diabetes Strategic Plan (NIDDK 2011) acknowledged the growing science base in this area and cited the need to understand more about the role of environmental exposures as part of future research and prevention strategies. To help develop such a research strategy, the National Toxicology Program (NTP) at the National Institute of Environmental Health Sciences (NIEHS) organized a state-of-the-science workshop in January 2011 titled “Role of Environmental Chemicals in the Development of Diabetes and Obesity” (NTP 2011). The objective of this workshop was to examine the literature for evidence of associations between certain chemicals and obesity or diabetes. Epidemiological studies of associations between diabetes and POPs, particularly the halogenated POPs, were considered at the workshop, along with studies of diabetes in association with arsenic, maternal smoking during pregnancy, bisphenol A, phthalates, organotins, and nonpersistent pesticides (Thayer et al. 2012). A wide variety of chemicals were included in the POPs category, including organochlorines [2,3,7,8-tetrachlorodibenzo-*p*-dioxin (TCDD or dioxin), Agent Orange, other non-TCDD polychlorinated dibenzo-*p*-dioxins (PCDDs), polychlorinated dibenzofurans (PCDFs), polychlorinated biphenyls (PCBs), dichlorodiphenyltrichloroethane (DDT), dichlorodiphenyldichloroethylene (DDE), and dichlorodiphenyldichloroethane (DDD)]; brominated compounds [polybrominated diphenyl ethers (PBDEs) and polybrominated biphenyls (PBBs)]; and perfluorinated compounds [perfluorooctane sulfonate (PFOS), perfluorooctanoic acid (PFOA), perfluorohexane sulfonate, and perfluorononanoic acid].

For the present review we evaluated the literature in terms of consistency, strengths and weaknesses (including power and statistical methods) of the clinical diagnosis, exposure assessment, and study population characteristics in order to identify data gaps and areas for future evaluation and research in the area of POPs exposure and diabetes outcomes.

## Methods

*Literature search*. We developed a PubMed (http://www.ncbi.nlm.nih.gov/pubmed) Medical Subject Headings (MeSH)-based and keyword search–based strategy to identify epidemiological studies of POPs exposure (organochlorine, organofluorine, and organobromine compounds) and health outcomes related to T1D, T2D, and childhood obesity [for detailed information on the literature search strategy, see Supplemental Material, pp. 2–3 (http://dx.doi.org/10.1289/ehp.1205502)]. We conducted an initial search on 24 August 2009 and subsequently updated the search through 15 December 2010. Studies of POPs and T2D or diabetes-related outcomes (e.g., metabolic syndrome) in both adults and children were eligible for review. We excluded studies from consideration if they were occupational studies, used death certificates to identify T2D, or did not present original data. Because of time constraints, we formally assessed only studies with T2D as the outcome, excluding studies with metabolic syndrome as the outcome. Our search identified 2,752 publications (after removal of duplicates), 72 of which presented original data on diabetes-related studies (see Supplemental Material, Figure S1). We excluded 28 studies from consideration because the health outcome was not T2D or because the method used to measure exposure or classify T2D was not adequate (see Supplemental Table S1). We considered blood or target tissue levels the most informative exposure measures; however, this information was not always available (e.g., studies of Vietnam veterans). Studies on Vietnam veterans were excluded if they were not specific enough to imply exposure to Agent Orange or TCDD; for example, studies comparing veterans who were in Vietnam with those who were not in Vietnam were excluded because they did not specify exposed versus unexposed veterans. We did not consider occupational studies because exposure may be more targeted depending on the occupation, nor did we consider a study by Anderson-Mahoney et al. (2008) because the population studied comprised plaintiffs involved in a lawsuit filed due to unusally high PFOA levels in drinking water. In addition, we chose to limit the introduction of potential biases that are unique to these studies, such as the healthy worker effect. We also excluded studies that used death certificates to identify diabetes cases because the prevalence of diabetes is underestimated from mortality data. For example, in a U.S-based study that characterized the sensitivity and specificity of death certificates for diabetes (Cheng et al. 2008), diabetes was listed as a direct or contributing cause of death on only 6.2% of the death certificates for adults who were known to have diabetes.

We identified an additional 17 articles by reviewing the reference lists in the primary literature and review articles, for a total of 43 studies.

*Data extraction*. NTP Office of Health Assessment and Translation staff extracted the main findings from the included studies [see Supplemental Material, Table S2 (http://dx.doi.org/10.1289/ehp.1205502)]. The identification of the main findings was based on the following strategy:

When a study did not report a statistically significant association (i.e., *p* > 0.05) between POPs exposure and T2D at any exposure level, we extracted the main finding from the highest exposure group compared with the referent group (e.g., fourth quartile vs. first quartile).
When a study reported a statistically significant association (i.e., *p* ≤ 0.05) between POPs exposure and T2D and that association displayed a monotonic dose response, we extracted the main finding based on the lowest exposure group with a statistically significant association (e.g., third quartile vs. first quartile).
When associations were nonmonotonic in nature, we identified the main findings on a case-by-case basis and considered any statistical trend analyses that might have been conducted, consistency of the overall pattern across exposure groups, and/or the biological significance of the nonmonotonic finding.

POPs represent a toxicologically diverse range of chemicals, all of which are persistent in the body (i.e., have a long half-life) and the environment. Chemicals are broadly divided into categories based on the halogen group (e.g., chlorinated, fluorinated, brominated). Chemicals in the chlorinated group were further divided into common chemical class designations (i.e., dioxins, PCBs, DDT/DDE/DDD). In assessing the PCB studies, we evaluated both total PCBs and PCB153 together because PCB153 is a major contributor to total PCB exposure and is used as an indicator PCB. PCB153 is often used as a surrogate measure for total PCBs because it is less expensive to measure (Cote et al. 2006; Meeker and Hauser 2010). Assessing patterns of association for individual PCBs across studies is particularly challenging because the class contains 209 structures that are not easy to categorize on the basis of structural similarity and/or biological activity. Even the categorization of “dioxin-like” or “nondioxin-like” is not sufficient because both categories of PCBs are linked to diabetes (Giesy and Kannan 1998; Lee et al. 2006, 2010, 2011a). In general, the findings for individual PCB congeners other than PCB153 are less suggestive for an overall association [see Supplemental Material, Figure S2 (http://dx.doi.org/10.1289/ehp.1205502)] (Codru et al. 2007; Everett et al. 2007; Lee et al. 2010; Patel et al. 2010; Turyk et al. 2009a).

**Figure 1:**
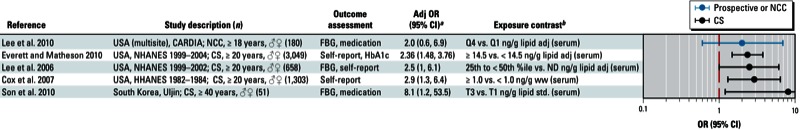
Associations between *trans*-nonachlor and diabetes in epidemiological studies. Abbreviations: %ile, percentile; Adj, adjusted; CARDIA, Coronary Artery Risk Development in Young Adults; CS, cross-sectional; FBG, fasting blood glucose; HbA1c, glycated hemoglobin; HHANES, Hispanic Health and Nutrition Examination Survey; NCC, nested case–control; ND, not determined; NHANES, National Health and Nutrition Examination Survey; Q, quartile; std, standardized; T, tertile; ww, wet weight. Self-report indicates a self-reported diagnosis of T2D; medication refers to medications used to treat T2D; and FBG and HbA1c indicate levels that were sufficiently elevated to be classified as T2D. ^*a*^Values are adjusted ORs unless otherwise noted. ^*b*^If no lipid adjustments were reported, the OR was not lipid adjusted; all exposures were measured in serum samples.

*Study quality*. We categorized studies into groups on the basis of study design and nature of the exposure: *a*) cohort studies with a prospective or nested case–control design, *b*) cross-sectional studies, *c*) case–control studies, *d*) occupational studies, *e*) ecological studies, *f* ) studies of maternal exposure, and *g*) studies of Vietnam veterans.

We included a study for consideration if it identified T2D as the outcome and the exposure measure was deemed adequate. Study quality was evaluated by panel members during workshop deliberations. Aspects of study quality included potential selection bias, possibility of association resulting from reverse causation, or loss to follow-up. These aspects were not summarized for each study but were considered during the discussion.

**Figure 2:**
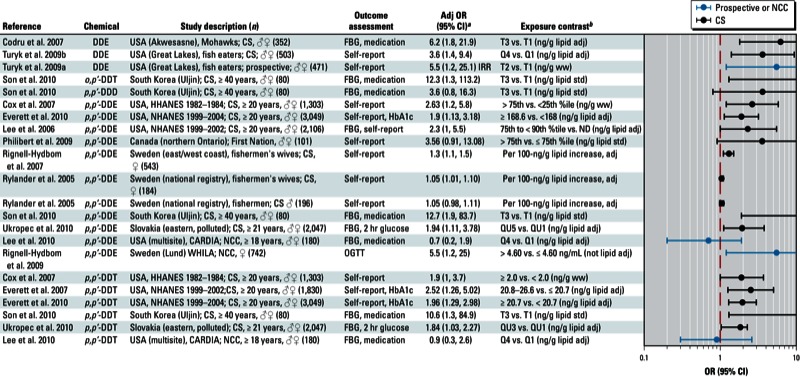
Association between DDE, DDT, or DDD and diabetes in epidemiological studies. Abbreviations: %ile, percentile; Adj, adjusted; CARDIA, Coronary Artery Risk Development in Young Adults; CS, cross-sectional; FBG, fasting blood glucose; HbA1c, glycated hemoglobin; HHANES, Hispanic Health and Nutrition Examination Survey; IRR, incidence rate ratio; ND, not determined; NCC, nested case–control; NHANES, National Health and Nutrition Examination Survey; OGTT, oral glucose tolerance test; Q, quartile; QU, quintile; std, standardized; T, tertile; ww, wet weight. Self report indicates self-reported diagnosis of T2D; medication refers to medications used to treat T2D; and OGTT, FBG, and HbA1c indicate levels that were sufficiently elevated to be classified as T2D. ^*a*^Values are adjusted ORs unless otherwise noted. ^*b*^If no lipid adjustments were reported, the OR was not lipid adjusted; all exposures were measured in serum samples.

*Use of Meta Data Viewer to assess patterns of findings*. The POPs literature on diabetes is quite complex, consisting of 72 epidemiological studies that often reported findings for multiple compounds in the same study. To visually assess patterns of primary study findings from this literature, we used a newly developed software program, the Meta Data Viewer (Boyles et al. 2011). In brief, the Meta Data Viewer is a graphing program that can display up to 15 text columns and graph 1–10 numerical values. The input data file is an Excel document, and users can sort, group, and filter data to look at patterns of findings across studies. We used this software program to visually display data during the workshop and to generate the figures presented below. The odds ratios (ORs) and 95% confidence intervals (CIs) are presented as they were reported by the study’s authors; in some cases, rounding may affect the appearance of symmetry for the 95% CIs. The graphing program, accompanying data file, and instructions for use are publicly accessible from the NTP (http://ntp.niehs.nih.gov/go/tools_metadataviewer). The data file currently contains 870 main findings from > 200 human studies on diabetes- and childhood obesity–related outcomes for POPs, as well as other exposures such as metals (e.g., arsenic, cadmium, lead, mercury), bisphenol A, nonpersistent pesticides, phthalates, and maternal smoking during pregnancy. Meta Data Viewer is a public resource; the program and any associated NTP data files are available for research and publication.

*Main findings*. We took into account patterns of findings for chemicals or chemical classes if at least three different studies reported diabetes-related outcomes for that chemical or chemical class. We did not consider epidemiological evidence sufficient to determine whether any of the positive associations were causal in nature.

**Figure 3:**
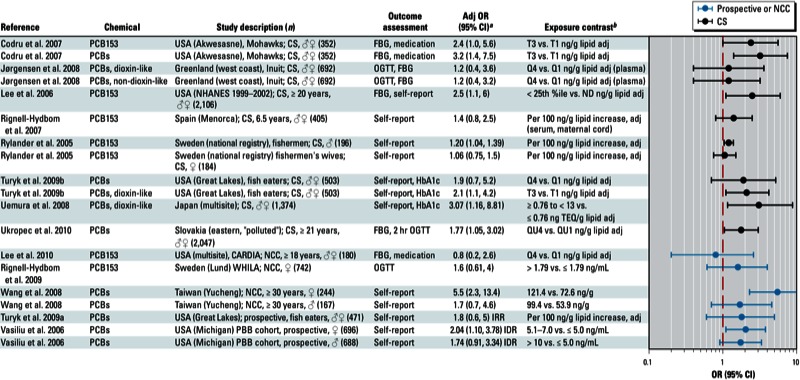
Association between PCBs and diabetes in epidemiological studies. Abbreviations: %ile, percentile; Adj, adjusted; CARDIA, Coronary Artery Risk Development in Young Adults; CS, cross-sectional; FBG, fasting blood glucose; HbA1c, glycated hemoglobin; IDR, incidence density ratio; IRR, incidence rate ratio; ND, not determined; OGTT, oral glucose tolerance test; Q, quartile; T, tertile; WHILA, Women’s Health in the Lund Area. Self-report indicates self-reported diagnosis of T2D; medication refers to medications used to treat T2D; and OGTT, FBG, and HbA1c indicate levels that were sufficiently elevated to be classified as T2D. ^*a*^Values are adjusted ORs unless otherwise noted. ^*b*^If no lipid adjustments were reported, the OR was not lipid adjusted; exposures were measured in serum samples unless otherwise indicated.

**Figure 4:**
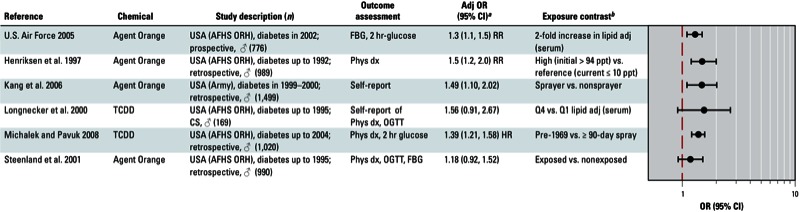
Association between Agent Orange or dioxin and diabetes in studies of Vietnam veterans. Abbreviations: Adj, adjusted; AFHS ORH, Air Force Health Study, Operation Ranch Hand; FBG, fasting blood glucose; OGTT, oral glucose tolerance test; Phys dx, physcian diagnosis; HR, hazard ratio; RR, relative risk; Q, quartile; OGTT and FBG indicate levels that were sufficiently elevated to be classified as T2D. ^*a*^Values are adjusted ORs unless otherwise noted. ^*b*^If no lipid adjustments were reported, the OR was not lipid adjusted.

The strongest positive associations were with *trans*-nonachlor (Figure 1); DDE, DDT, and DDD (Figure 2); dioxins/dioxin-like chemicals and certain PCBs (Figure 3); and Agent Orange or TCDD in Vietnam veterans (Figure 4). Findings from studies of *trans*-nonachlor (Airaksinen et al. 2011; Lee et al. 2011a), DDE (Airaksinen et al. 2011; Grandjean et al. 2011; Lee et al. 2011a), and PCBs (Grandjean et al. 2011; Lee et al. 2011a; Persky et al. 2011) published after the workshop are consistent with the conclusions reached during the workshop [see Supplemental Material, Figures S2 and S3 (http://dx.doi.org/10.1289/ehp.1205502)].

**Figure 5:**
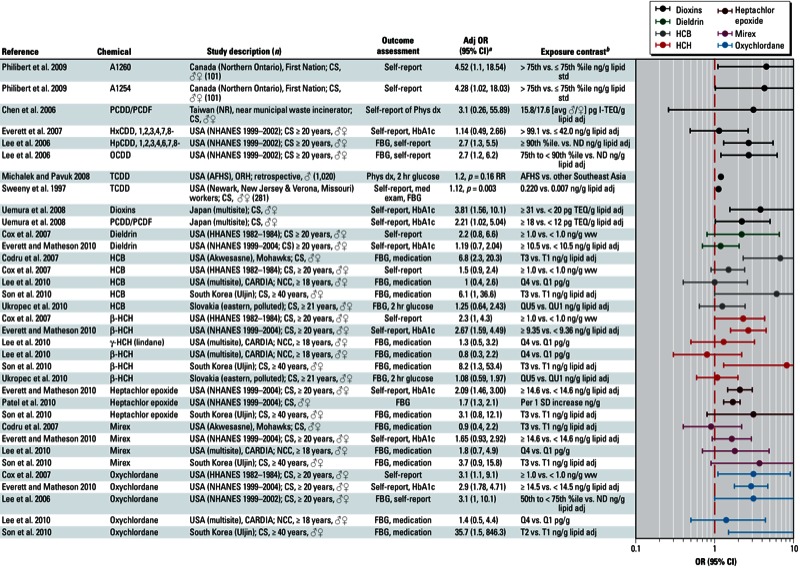
Association between miscellaneous organochlorine POPs and diabetes in epidemiological studies. Abbreviations: %ile, percentile; Adj, adjusted; AFHS ORH, Air Force Health Study, Operation Ranch Hand; avg, average; CARDIA, Coronary Artery Risk Development in Young Adults; CS, cross-sectional; FBG, fasting blood glucose; HbA1c, glycated hemoglobin; HHANES, Hispanic Health and Nutrition Examination Survey; I‑TEQ, international toxic equivalent; med exam, medical exam; NCC, nested case–control; ND, not determined; NHANES, National Health and Nutrition Examination Survey; OGTT, oral glucose tolerance test; Phys dx, physician diagnosis; PIVUS, Prospective Investigation of the Vasculature in Uppsala Seniors; Q, quartile; QU, quintile; RR, relative risk; std, standardized; T, tertile; TEQ, toxic equivalents; ww, wet weight. Self-report indicates self-reported diagnosis of T2D; medication refers to medications used to treat T2D; and OGTT, FBG, and HbA1c indicate levels that were sufficiently elevated to be classified as T2D. ^*a*^Values are adjusted ORs unless otherwise noted. ^*b*^If no lipid adjustments were reported, the OR was not lipid adjusted; exposures were measured in serum samples unless otherwise indicated.

**Figure 6:**
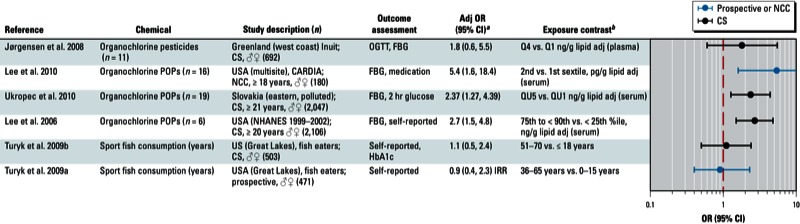
Association between POPs mixtures and diabetes in epidemiological studies. Abbreviations: %ile, percentile; Adj, adjusted; CARDIA, Coronary Artery Risk Development in Young Adults; CC, case–control; CS, cross-sectional; FBG, fasting blood glucose; HbA1c, glycated hemoglobin; IRR, incidence rate ratio; NHANES, National Health and Nutrition Examination Survey; OGTT, oral glucose tolerance test; Q, quartile; QU, quintile; T, tertile. Self-report indicates self-reported diagnosis of T2D; medication refers to medications used to treat T2D; and OGTT, FBG, and HbA1c indicate levels that were sufficiently elevated to be classified as T2D. ^*a*^Values are adjusted ORs unless otherwise noted. ^*b*^If no lipid adjustments were reported, the OR was not lipid adjusted.

Among specific organochlorine chemicals that were evaluated in < 6 studies, including dieldrin, hexachlorobenzene (HCB), β-hexachlorocyclohexane (β-HCH), lindane (γ-HCH), heptachlor epoxide, mirex, and oxychlordane, we found positive patterns of associations (Figure 5). However, in many cases the estimates of association reported by individual studies were not statistically significant (Chen et al. 2006; Codru et al. 2007; Cox et al. 2007; Everett et al. 2007; Everett and Matheson 2010; Lee et al. 2006, 2010; Michalek and Pavuk 2008; Patel et al. 2010; Son et al. 2010; Steenland et al. 2001; Sweeney et al. 1997; Uemura et al. 2008; Ukropec et al. 2010). In a similar manner, an overall pattern of a positive association was apparent in studies of mixtures of organochlorine POPs (Jørgensen et al. 2008; Lee et al. 2006, 2010; Ukropec et al. 2010) (Figure 6).

**Figure 7:**
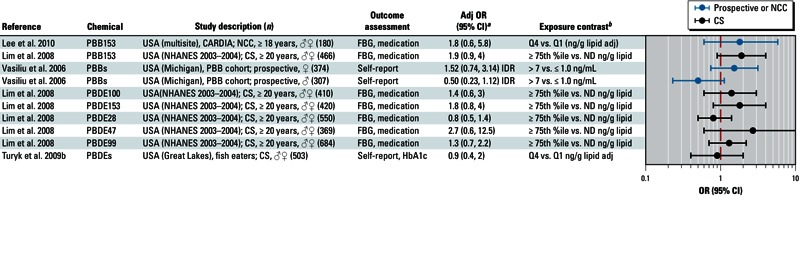
Association between brominated compounds and diabetes in epidemiological studies. Abbreviations: %ile, percentile; Adj, adjusted; CARDIA, Coronary Artery Risk Development in Young Adults; CC, case–control; CS, cross-sectional; FBG, fasting blood glucose; HbA1c, glycated hemoglobin; IDR, incidence density ratio; ND, not determined; NHANES, National Health and Nutrition Examination Survey; Q, quartile. Self-report indicates self-reported diagnosis of T2D; medication refers to medications used to treat T2D; and FBG and HbA1c indicate levels that were sufficiently elevated to be classified as T2D. ^*a*^Values are adjusted ORs unless otherwise noted. ^*b*^If no lipid adjustments were reported, the OR was not lipid adjusted; all exposures were measured in serum samples.

**Figure 8:**
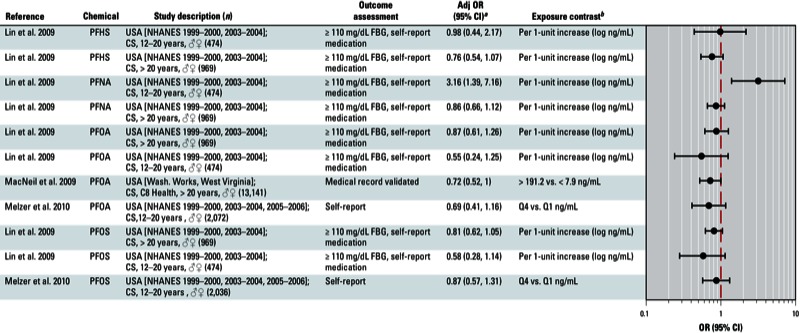
Association between perfluoro­alkyl acids and diabetes in epidemiological studies. Abbreviations: Adj, adjusted; C8 Health, C8 Health Project; CS, cross-sectional; FBG, fasting blood glucose; NHANES, National Health and Nutrition Examination Survey; PFHS, perfluoro­hexane sulfonate; PFNA, perfluoro­nonanoic acid; Q, quartile. Self-report indicates self-reported diagnosis of T2D. ^*a*^Values are adjusted ORs unless otherwise noted. ^*b*^If no lipid adjustments were reported, the OR was not lipid adjusted; all exposures were measured in serum samples.

Overall, we found that organochlorine compounds were positively associated with diabetes. Workshop participants concluded that there was not sufficient evidence for an association between T2D and PBBs or PBDEs (Lee et al. 2010; Lim et al. 2008; Turyk et al. 2009b; Vasiliu et al. 2006) (Figure 7). Results from studies examining an association between T2D and PBDE153 and PBDE47, which were published after the workshop, are consistent with this initial assessment [Airaksinen et al. 2011; Lee et al. 2011a; see also Supplemental Material, Figure S2 (http://dx.doi.org/10.1289/ehp.1205502)]. Workshop participants also concluded that evidence for an association between T2D and perfluoroalkyl acids, such as PFOS and PFOA, was not sufficient (Costa et al. 2009; Lin et al. 2009; MacNeil et al. 2009; Melzer et al. 2010; Nelson et al. 2010) (Figure 8).

## Discussion

The purpose of this evaluation was not only to assess the epidemiological literature for evidence of associations between POPS and T2D but also to collaboratively identify data gaps and areas for future research in the area of POPs exposure and outcomes related to diabetes. The resulting list of data gaps includes topics that are related to but not specifically discussed here. For example, we found only one epidemiological study on POPs and T1D, a very important health outcome (Rignell-Hydbom et al. 2010). The full list of data gaps and research needs recommended by workshop participants based on the literature review are summarized in Appendix 1.

*Vietnam veteran studies*. The conclusion from our evaluation, that there is an association between POPs and diabetes in Vietnam veterans, differs somewhat from assessments conducted by the Institute of Medicine (IOM) Committee to Review the Health Effects in Vietnam Veterans of Exposure to Herbicides (IOM 1994, 2001, 2011). The evidence for an association between exposure to herbicides used during the Vietnam War and long-term health effects in veterans, including diabetes, is assessed every other year by this committee as part of the Agent Orange Act of 1991. The strength-of-evidence conclusion from the epidemiological studies included in the first report (IOM 1994) was for “inadequate/insufficient evidence to determine whether an association exists” between exposure to herbicides [2,4-dichlorophenoxyacetic acid (2,4-D), 2,4,5-trichlorophenoxyacetic acid (2,4,5-T) and its contaminant TCDD, cacodylic acid, and Picloram] and diabetes mellitus. However, a committee convened by the IOM in 1999 to conduct a specific review of the scientific evidence regarding T2D and Agent Orange in Vietnam veterans concluded that there was limited or suggestive evidence of an association between T2D and exposure to Agent Orange used in Vietnam (IOM 2001). This conclusion was maintained in *The Veterans and Agent Orange* updates in 2001, 2002, 2004, 2006, 2008, and 2010 (IOM 2011). In contrast, our conclusion from the present evaluation is that there is evidence for a positive association when the data were considered collectively (Figure 4).

*Risk factors and confounding*. Epidemiological studies regarding POPs and diabetes and other metabolic disorders should consider sex, age, race/ethnicity, and combinations of exposures with other agents (e.g., plastic-associated compounds such as bisphenol A, metals) as potential confounding or modifying variables.

It is less clear whether studies should use lipid-standardized blood measurement for lipophilic chemicals; several different approaches are currently used in models, including *a*) wet concentrations without consideration of lipid profiles, *b*) lipid-standardized concentrations, or *c*) wet concentrations with lipid adjustment. Because POPs circulate with serum lipids, high blood lipids increase measured levels of POPs. Therefore, the failure to account for this relationship may result in the overestimation of relative risks. However, the exposure to certain chlorinated POPs can lead to increased levels of serum lipids, and dyslipidemia is involved in the pathogenesis of T2D, suggesting that dyslipidemia may be an intermediate factor in the relationship between POPs and T2D. In this situation, adjusting for this relationship may underestimate true associations. Even though true associations may be somewhere between unadjusted and adjusted results, there is uncertainty about the most appropriate way to deal with lipids.

Adjusting for obesity is controversial in studying the association between POPs and diabetes. There is growing evidence that obesity is on the causal pathway between POPs and diabetes (Lee et al. 2011b; Ruzzin et al. 2010). In addition, this relationship is potentially confounded by the consumption of fatty food, which is associated with obesity and with increased POPs levels. However, adipose tissue serves as a reservoir of POPs, thereby reducing the circulating POPs level (Lim et al. 2011). This effect might have a positive role in limiting the exposure to target tissues for diabetes, such as pancreatic β-cells.

*Nonmonotonic exposure–response relationships*. Several of the reviewed studies reported evidence of nonmonotonic exposure–response relationships. For example, in the CARDIA (Coronary Artery Risk Development in Young Adults) cohort, estimated associations with diabetes were strongest for the second quartile of exposure to *trans*-nonachlor, oxychlordane, mirex, highly chlorinated PCBs, and PBB153 (Lee et al. 2010). Other studies (Lee et al. 2011a; Rignell-Hydbom et al. 2009; Turyk et al. 2009a) reported monotonic relationships. A closer evaluation of the dose–response curves from each of these studies (Lee et al. 2011a; Rignell-Hydbom et al. 2009; Turyk et al. 2009a) revealed that the risk of diabetes was substantially increased with only small increases within the lower ranges of POPs concentrations, but only slightly increased with higher increases in concentrations of POPs. For example, in the PIVUS (the Prospective Investigation of the Vasculature in Uppsala Seniors) study, the adjusted ORs across quintiles of summary measures of PCBs were 1.0, 4.5, 5.1, 8.8, and 7.5 (Lee et al. 2011b).

In this sense, the dose–response curves presented in these studies share the low-dose portion of a wide inverted U-shaped association. Varying background exposure distributions may contribute to different forms of the concentration–response curves seen between studies, depending on the relative importance of different POPs in the background mixture. The inverted U-shaped association has been suspected to be biologically linked to the endocrine-disrupting properties of POPs because an increase from no to low occupancy of hormone receptors has been observed to have linear effects on hormone-mediated phenomena, but that effect sometimes decelerated or even stopped when the dose increased (Vandenberg et al. 2012). Thus, improving understanding of the biological basis for potential nonlinear relationships was considered by the workshop participants to be an important research need (Appendix 1).

*Meta-analysis or pooled analysis of existing studies*. The workshop participants discussed the possibility of conducting a meta-analysis of existing studies, or a pooled analysis of individual-level data from prospective studies, in particular the five prospective studies of PCB153 and DDE (Lee et al. 2010, 2011a; Rignell-Hydbom et al. 2009; Turyk et al. 2009a; Vasiliu et al. 2006). However, the participants concluded that there was too much variation across studies to permit a meta-analysis or pooled analysis. For example, the five studies of PCB153 and DDE mentioned above used different diagnostic strategies and approaches to address confounding, particularly by serum lipid levels (Lee et al. 2010). The cohorts also varied with regard to age, from 18 to 30 years (Lee et al. 2010) to 70 years (Lee et al. 2011a), and sex, which was exclusively female in one study (Rignell-Hydbom et al. 2009), exclusively male in another (Lee et al. 2010), and mixed in the remaining cohorts (Lee et al. 2011a; Turyk et al. 2009a; Vasiliu et al. 2006). In addition, temporal and geographic variation among the cohorts resulted in substantial differences in the chemical mixtures to which the populations were exposed as well as the duration and relative concentrations of exposures.

*Causality*. Although several organochlorine compounds showed positive associations with T2D, we cannot determine whether these associations are causal in nature based on observational epidemiologic studies alone; additional animal and *in vitro* mechanistic studies are needed to clarify the role of POPs in metabolic disease development. Factors to be considered in such studies should address the influence of time windows of exposure; exposure measurements (e.g., the chemical analysis of individual POPs); chemical mixtures identifying relevant tissue targets; biological mechanisms that lead to obesity, insulin resistance, lipidemia, and diabetes; and the influence of genetic variation among animal models. Combining results from relevant mechanistic and animal studies with findings from epidemiologic studies would enhance our ability to establish a possible causal linkage between POPs and diabetes.

Identification of individual chemicals or chemical mixtures that are associated with T2D in epidemiology studies will help direct further toxicity testing. The combined use of toxicity testing and screening of chemical classes using assays relevant to diabetes will also help epidemiologists determine which chemicals to measure in future studies. The structures of chemicals that are associated with diabetes are highly variable, and it is difficult to link them to a common etiologic mechanism. Further research to identify all relevant pathways to diabetes will aid in deciphering structure–activity relationships.

Although our evaluation focused on the epidemiological data, findings from *in vitro* and animal studies show that TCDD, PCBs, and other chlorinated POPs can cause pancreatic effects (Ebner et al. 1993; Rao et al. 1988; Rozman et al. 1986; Wassermann et al. 1975) and influence insulin signaling (Ibrahim et al. 2011; Kim et al. 2009; Nishiumi et al. 2010; Ruzzin et al. 2010; Tang et al. 2007; Wang et al. 2010), glucose-stimulated insulin secretion (Fischer et al. 1999; Hsu et al. 2010; Kurita et al. 2009; Novelli et al. 2005; Piaggi et al. 2007), glucose uptake (Enan et al. 1992a, 1992b; Olsen et al. 1994), gluconeogenesis (Boll et al. 1998; Gorski et al. 1990; Viluksela et al. 1999), and adipocyte differentiation or regulation (Arsenescu et al. 2008; Hsu et al. 2010; Mullerova and Kopecky 2007; Shimba et al. 2001).

However, the laboratory animal data on organochlorine-induced changes in glucose and insulin levels are not necessarily consistent with associations between POPs and an increased incidence of T2D reported by epidemiologic studies (Everett et al. 2007; Uemura et al. 2008). It is unclear whether the lack of consistency results from physiological differences between rodents and humans in the development of diabetes, or from experimental variables related to differences in exposure levels, the window of exposure, and/or the duration of exposure and length of follow-up. Much of the work in this area is based on TCDD exposure. In humans, diabetes is characterized by increased blood glucose levels. In contrast, in different animal models, TCDD has been shown to cause hypoglycemia (Fried et al. 2010; Gorski and Rozman 1987; Viluksela et al. 1998, 1999), to have no effect on glucose levels (Unkila et al. 1995), or to cause both hyperglycemia and hypoglycemia at different time points during or after dosing (Ebner et al. 1988; Potter et al. 1983). Although epidemiology studies tend to show a positive relationship between TCDD body burdens and insulin levels (Cranmer et al. 2000; Michalek et al. 1999), TCDD typically causes hypoinsulinemia and increased insulin sensitivity in animals (Ebner et al. 1988; Fried et al. 2010; Gorski et al. 1988; Gorski and Rozman 1987; Potter et al. 1983; Stahl et al. 1992; Weber et al. 1987). Thus, in animal models, exposure to TCDD mimics the feature of reduced insulin secretion observed in the clinical progression of prediabetes to overt diabetes. Inhibition of glucose uptake may at least partially explain why hypoinsulinemia is frequently observed in animal studies. In most tissues studied, TCDD inhibits glucose uptake by decreasing the activity or protein level of glucose transporter (GLUT) proteins responsible for transporting blood glucose to adipose, muscular, pancreatic, hepatic, and intestinal epithelial tissue (El-Sabeawy et al. 2001; Enan et al. 1992b; Liu and Matsumura 1995; Matsumura 1995; Olsen et al. 1994). Decreased glucose uptake into the pancreas could mean that pancreatic β-cells do not sense higher blood glucose levels and therefore do not elicit an insulin response to those levels (Matsumura 1995). The level of glucose-uptake inhibition appears to correlate with the activation of the aryl hydrocarbon receptor, which is required for TCDD-induced toxicological effects (Matsumura 1995; Olsen et al. 1994). However, the dioxin exposures in these *in vivo* and *in vitro* studies are approximately 1,000–100,000 times background body burdens observed in the U.S. population. The *in vivo* studies are associated with body weight loss, histopathological findings, and significant decreases in thyroid hormones. Extrapolating these effects and mechanisms to background human exposures is challenging.

## Conclusions

Diabetes is a major threat to public health worldwide (WHO 2011); although there are well-established risk factors for diabetes (e.g., excess weight), environmental chemicals might also contribute to the etiology of this disease. On the basis of our review of human epidemiological studies, we conclude that there is support for positive associations between diabetes and certain chlorinated POPs. We identified a number of research needs (Appendix 1), noting in particular the need to *a*) better understand the relationships between both developmental and adult exposure to POPs and obesity, diabetes, and related metabolic disturbances; *b*) identify mechanisms for the observed associations, which will require basic research to develop better animal models and identify relevant biological pathways that could be assessed using *in vitro* screening systems; *c*) understand the modifying effects of factors such as inflammation, visceral fat, other chemical exposures, genotype, age at exposure, and the duration of exposure; and *d*) develop improved methods to measure POPs in small blood volumes using high throughput technologies at a reasonable cost.

T2D is a debilitating disease that affects adults as well as children and adolescents. The economic impact of the disease is enormous, not only in terms of direct medical costs but also on lost productivity. Therefore, understanding the impact of environmental factors such as chemical exposures is a high-priority research goal (NIDDK 2011). Exposure to environmental chemicals may be an additional risk factor that, if prevented, could facilitate a reduction in disease incidence and in the overall associated health and economic burden.

## Appendix

**Data gaps and research recommendations**

**Data gaps:**

The effects of mixtures on POPs and other environmental chemicals
High throughput surrogate exposure measures based on biological activity
Longitudinal studies with repeated measurements of developmental exposures and outcomes (e.g., obesity, diabetes, related metabolic disturbances) to follow progression of disease
Relationships between POPs and T1D [only one prospective study (Rignell-Hydbom et al. 2010)]
Studies on age, time period, and cohort effects of POPs exposure and incident diabetes
Studies of T2D in non­overweight or obese individuals.

**Research recommendations:**

Promote collaboration between epidemiologists, clinicians, and laboratory scientists to work in a true translational way
Perform epidemiological and animal studies of the progressive development of disease over time considering factors such as genetics, age, window of exposure, and lifestyle
Develop better animal models of diabetes and obesity
Include measurement of glucose end points, lipid profiles, insulin resistance, waist circumference and other measures of obesity, and blood pressure in studies
Include the interaction between POPs exposure and genotype in regard to future T1D and T2D diabetes development
Improve understanding of non­monotonic relationships (i.e., frequency of occurrence and biological basis)
Focus on chemicals present in the current population for which the extent of exposure is expected to increase or stay the same
Consider differences in exposure across generations
Consider the influence of subclinical disease on biomarkers of exposure
Develop improved high throughput assays to measure POPs in low blood volumes at a reasonable cost
Use improved analytical measures on biobanked blood from existing longitudinal studies
Identify biological pathways for diabetes and related disease states, and screen existing POPs for activity in these pathways in high throughput assay systems.

## Supplemental Material

(1.3 MB) PDF

(37 KB) XSLX

(66 KB) XLSX
