# Supplementary material for: Evaluation of the Association between Persistent Organic Pollutants (POPs) and Diabetes in Epidemiological Studies: A National Toxicology Program Workshop Review
Source: Environ Health Perspect. 2013 May 7;121(7):774–83. doi: 10.1289/ehp.1205502 (PMC3701910; doi:10.1289/ehp.1205502)
Supplement: (1.3 MB) PDF [file ehp.1205502.s001.pdf]

Supplemental Materials
Evaluation of the Association between Persistent Organic Pollutants (POPs) and Diabetes in Epidemiological Studies: A National Toxicology Program Workshop Review

Kyla W. Taylor, Raymond F. Novak, Henry A. Anderson, Linda S. Birnbaum, Chad Blystone, Michael DeVito, David Jacobs, Josef Köhrle, Duk-Hee Lee, Lars Rylander, Ana Rignell-Hydbom, Rogelio Tornero-Velez, Mary E. Turyk, Abee L. Boyles, Kristina A. Thayer, and Lars Lind

Table of Contents

Supplemental Material, Literature search strategy 2

Supplemental Material, Table S1. Summary of diabetes studies excluded from the January 2011 workshop. 3

Supplemental Material, Table S2. Summary of studies included in the January 2011 workshop...3

Supplemental Material, Figure S1. Flow diagram of study identification and exclusions for studies considered up to 12-15-2010 4

Supplemental Material, Figure S2. Main findings from studies of individual PCB congeners, other than PCB153, published prior to January 2011 workshop 5

Supplemental Material, Figure S3. Main findings from PCB studies published subsequent to January 2011 workshop 6

References Cited 8

Supplemental Material, Literature search strategy

MeSH-based PubMed search: (("Polychlorinated Biphenyls"[Mesh] OR "Hydrocarbons, Chlorinated"[Mesh] OR "Dioxins"[Mesh] OR "Halogenated Diphenyl Ethers"[Mesh] OR "Polybrominated Biphenyls"[Mesh] OR "perfluorooctane sulfonic acid"[Substance Name] OR "perfluorooctanoic acid"[Substance Name]) AND (("obesity"[mh] OR "body mass index"[mh] OR "weight gain"[mh] OR "adipogenesis"[mh] OR "adipose tissue"[mh] OR "adipokines"[mh] OR "adiponectin"[mh] OR "leptin"[mh] OR resistin[mh]) OR ("diabetes mellitus"[mh] OR "glucose metabolism disorders"[mh] OR "insulin"[mh] OR "insulin resistance"[mh] OR "blood glucose"[mh] OR "islets of langerhans"[mh]))

Keyword-strategy to search "new" un-indexed articles: (("Polychlorinated Biphenyls" OR "chlorinated hydrocarbons" OR aldrin OR chlordane OR chlordecone OR chlorobenzene* OR hexachlorobenzene OR chloroform OR ddt OR dichlorodiphenyltrichloroethane OR dichloroacetate OR "dichlorodiphenyl dichloroethylene" OR dichlorodiphenyldichloroethane OR dichloroethylenes OR dieldrin OR endrin OR "ethyl chloride" OR "ethylene dichlorides" OR heptachlor OR lindane OR hexachlorocyclohexane OR methoxychlor OR "methyl chloride" OR "methylene chloride" OR mirex OR mitotane OR "picryl chloride" OR polychloroterphenyl OR tetrachloroethylene OR toxaphene OR trichloroepoxypropane OR trichloroethane* OR trichloroethylene OR "vinyl chloride" OR "Dioxins" OR TCDD OR "Halogenated Diphenyl Ethers" OR "diphenyl ethers" OR PBDE* OR PCDE* OR "Polybrominated Biphenyls" OR "polybrominated biphenyls" OR Polybromobiphenyl* OR "polychlorinated b iphenyls" OR Polychlorobiphenyl OR PCB OR "perfluorooctane sulfonic acid" OR "perfluorooctane sulfonic acid" OR pfosa OR 1763-23-1 OR "perfluorooctane sulfonate" OR "perfluorooctanoic acid" OR 335-67-1 OR "perfluorooctanoic acid" OR PFOA OR "pentadecafluorooctanoic acid" OR

"perfluorooctanoyl chloride" OR "sodium perfluorooctanoate" OR "perfluorinated octanoic acid") AND ((diabetes OR "glucose tolerance" OR "glucose intolerance" OR hyperglycemia OR hypoglycemia OR insulin OR "blood glucose" OR "metabolic syndrome" OR "syndrome x" OR "islets of langerhans") OR (obes* OR "body mass index" OR "body fat" OR "weight gain" OR adipos* OR adipogen* OR adipokine* OR leptin OR resistin OR adiponectin*)) AND (publisher[sb] OR "in process"[sb]))

Supplemental Material, Table S1. Summary of diabetes studies excluded from the January 2011 workshop.

[See separate Excel file]

Supplemental Material, Table S2. Summary of studies included in the January 2011 workshop

[See separate Excel file]

Supplemental Material, Figure S1. Flow diagram of study identification and exclusions for studies considered up to 12-15-2010

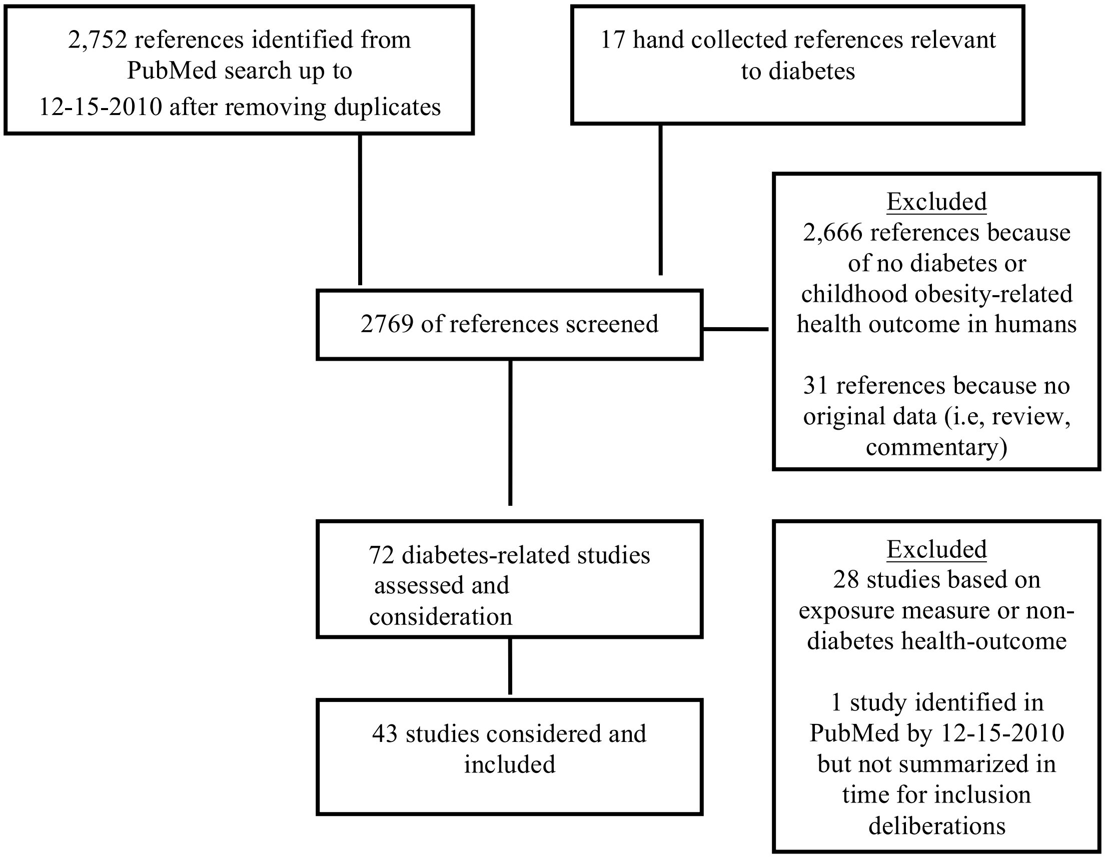

Supplemental Material, Figure S2. Main findings from studies of individual PCB congeners, other than PCB153, published prior to January 2011 workshop

Reference	Chemical	Study Description (n)	Outcome Assesment	adjOR (95% CI)a	Exposure Contrastb
Codru et al. 2007	PCB74	USA (NHANES 1999-2002), ≥ 20 years, ♂♀, CS (1,830)	FBG, meds	4.5 (1.3, 15.6)	T3 vs. T1 ng/g lipid adj.
Everett et al. 2007	PCB126	USA (NHANES 1999-2002) ≥20, ♂♀ CS (1,830)	SR, HbA1c	2.57 (1.33, 4.95)	>83.8 vs. ≤31.2 ng/g lipid adj.
Lee et al. 2010	PCB105	USA (multisite), CARDIA; NCC, ≥ 18 years, ♂♀ (180)	FBG, meds	0.2 (0.1, 0.8)	Q4 vs. Q1 pg/g
Lee et al. 2010	PCB118	USA (multisite), CARDIA; NCC, ≥ 18 years, ♂♀ (180)	FBG, meds	0.5 (0.2, 1.4)	Q4 vs. Q1 pg/g
Lee et al. 2010	PCB138-158	USA (multisite), CARDIA; NCC, ≥ 18 years, ♂♀ (180)	FBG, meds	0.8 (0.3, 2.2)	Q4 vs. Q1 pg/g
Lee et al. 2010	PCB146	USA (multisite), CARDIA; NCC, ≥ 18 years, ♂♀ (180)	FBG, meds	0.9 (0.3, 2.6)	Q4 vs. Q1 pg/g
Lee et al. 2010	PCB156	USA (multisite), CARDIA; NCC, ≥ 18 years, ♂♀ (180)	FBG, meds	0.8 (0.2, 2.9)	Q4 vs. Q1 pg/g
Lee et al. 2010	PCB157	USA (multisite), CARDIA; NCC, ≥ 18 years, ♂♀ (180)	FBG, meds	0.5 (0.1, 1.7)	Q4 vs. Q1 pg/g
Lee et al. 2010	PCB167	USA (multisite), CARDIA; NCC, ≥ 18 years, ♂♀ (180)	FBG, meds	0.5 (0.2, 1.3)	Q4 vs. Q1 pg/g
Lee et al. 2010	PCB170	USA (multisite), CARDIA; NCC, ≥ 18 years, ♂♀ (180)	FBG, meds	0.9 (0.3, 3.4)	Q4 vs. Q1 pg/g
Lee et al. 2010	PCB178	USA (multisite), CARDIA; NCC, ≥ 18 years, ♂♀ (180)	FBG, meds	2.7 (1, 7)	Q2 vs. Q1 pg/g
Lee et al. 2010	PCB180	USA (multisite), CARDIA; NCC, ≥ 18 years, ♂♀ (180)	FBG, meds	2.8 (1, 7.6)	Q2 vs. Q1 pg/g
Lee et al. 2010	PCB183	USA (multisite), CARDIA; NCC, ≥ 18 years, ♂♀ (180)	FBG, meds	0.8 (0.3, 2.3)	Q4 vs. Q1 pg/g
Lee et al. 2010	PCB187	USA (multisite), CARDIA; NCC, ≥ 18 years, ♂♀ (180)	FBG, meds	2.8 (1.1, 7.4)	Q2 vs. Q1 pg/g
Lee et al. 2010	PCB194	USA (multisite), CARDIA; NCC, ≥ 18 years, ♂♀ (180)	FBG, meds	0.4 (0.1, 1.5)	Q4 vs. Q1 pg/g
Lee et al. 2010	PCB195	USA (multisite), CARDIA; NCC, ≥ 18 years, ♂♀ (180)	FBG, meds	0.6 (0.2, 1.9)	Q4 vs. Q1 pg/g
Lee et al. 2010	PCB196-203	USA (multisite), CARDIA; NCC, ≥ 18 years, ♂♀ (180)	FBG, meds	0.6 (0.2, 2.2)	Q4 vs. Q1 pg/g
Lee et al. 2010	PCB199	USA (multisite), CARDIA; NCC, ≥ 18 years, ♂♀ (180)	FBG, meds	0.9 (0.3, 3)	Q4 vs. Q1 pg/g
Lee et al. 2010	PCB206	USA (multisite), CARDIA; NCC, ≥ 18 years, ♂♀ (180)	FBG, meds	0.5 (0.1, 1.6)	Q4 vs. Q1 pg/g
Lee et al. 2010	PCB209	USA (multisite), CARDIA; NCC, ≥ 18 years, ♂♀ (180)	FBG, meds	0.4 (0.1, 1.3)	Q4 vs. Q1 pg/g
Lee et al. 2010	PCB74	USA (multisite), CARDIA; NCC, ≥ 18 years, ♂♀ (180)	FBG, meds	2.8 (1, 7.3)	Q4 vs. Q1 pg/g
Lee et al. 2010	PCB87	US (multi-site) CARDIA ≥18y, ♂♀ nested CC (180)	FBG, meds	0.6 (0.2, 1.6)	Q4 vs. Q1 pg/g
Lee et al. 2010	PCB99	US (multi-site) CARDIA ≥18y, ♂♀ nested CC (180)	FBG, meds	0.8 (0.3, 2)	Q4 vs. Q1 pg/g
Patel et al. 2010	PCB170	USA (NHANES 1999-2004) ♂♀ CS (2,591)	FBG	2.2 (1.6, 3.2) per 1 SD	0.02-0.13[lo-hi range] ng/g
Philibert et al. 2009	PCB153	Canada (Northern Ontario) First Nation, ♂♀ (101)	SR	6.46 (2.07, 36.63)	>75th vs. ≤ 75th %tile, ng/g lipid stand.
Philibert et al. 2009	PCB74	Canada (Northern Ontario) First Nation, ♂♀ (101)	SR	6.06 (1.21, 30.27)	>75th vs. ≤ 75th %tile, ng/g lipid stand.
Philibert et al. 2009	PCBs	Canada (Northern Ontario) First Nation, ♂♀ (101)	SR	5.51 (1.26, 24.07)	>75th vs. ≤ 75th %tile, ng/g lipid stand.
Turyk et al. 2009a	PCB118	USA (Great Lakes) fish eaters, ♂♀ Pros (471)	SR	1.3 (0.5, 5) IRR	0.3-4.6 (T3) vs <2.2 (T1) ng/g wet weight

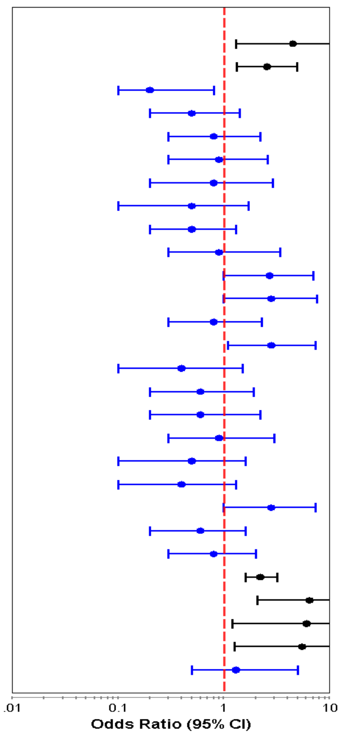

prospective or nested CC   cross-sectional

Abbreviations: CARDIA, Coronary Artery Risk Development in Young Adults Study; SR, self-reported type 2 diabetes diagnosis; Pros, prospective; NCC, nested case control; CS, cross-sectional; IRR, incidence rate ratio; FBG, fasting blood glucose ; meds, medications used to treat type 2 diabetes; OGTT, glucose tolerance test; HbA1c, Glycated haemoglobin; FBG, HbA1c, 2hr glucose, levels are sufficiently elevated to be classified as type 2 diabetes; SD, standard deviation; %ile, percentile; Q, quartile; T, tertile. [a]Values are adjusted ORs unless otherwise noted. [b]If no lipid adjustments were reported, the OR was not lipid adjusted; all exposures were measured in serum samples.

Supplemental Material, Figure S3. Main findings from PCB studies published subsequent to January 2011 workshop

Reference	*Chemical*	*Study Description (n)*	*Outcome Assessmentadj*	*OR (95% CI)a*	*Exposure Contrastb*
Grandjean et al. 2011	PCBs	Faroe Islands (residents' registry) 70-74 years, ♂♀ CS (1,131)	previous. dx, FGB, HbA1c	1.11 (0.91, 1.35)	Per 2x increase
Airaksinen et al. 2011	PCB153	Finland (Helsinki), 57-70 years , ♂♀, CS (1,988)	OGTT	1.64 (0.92, 2.93)	≥90th vs. <10th %ile ng/g lipid
Tanaka et al. 2011	PCB74	Japan [Saku] SCOP, ♂♀ CS (117)	FBG, HbA1c, meds	1.16 (0.68, 1.98)	NR
Tanaka et al. 2011	PCB99	Japan [Saku] SCOP, ♂♀ CS (117)	FBG, HbA1c, meds	1.70 (0.60, 4.80)	NR
Tanaka et al. 2011	PCB118	Japan [Saku] SCOP, ♂♀ CS (117)	FBG, HbA1c, meds	0.79 (0.54, 1.15)	NR
Tanaka et al. 2011	PCB138	Japan [Saku] SCOP, ♂♀ CS (117)	FBG, HbA1c, meds	1.17 (0.67, 2.06)	NR
Tanaka et al. 2011	PCB146	Japan [Saku] SCOP, ♂♀ CS (117)	FBG, HbA1c, meds	29.2 (1.89, 451)	NR
Tanaka et al. 2011	PCB153	Japan [Saku] SCOP, ♂♀ CS (117)	FBG, HbA1c, meds	0.73 (0.51, 1.07)	NR
Tanaka et al. 2011	PCB156	Japan [Saku] SCOP, ♂♀ CS (117)	FBG, HbA1c, meds	0.94 (0.12, 7.62)	NR
Tanaka et al. 2011	PCB163/164	Japan [Saku] SCOP, ♂♀ CS (117)	FBG, HbA1c, meds	0.14 (0.03, 0.58)	NR
Tanaka et al. 2011	PCB170	Japan [Saku] SCOP, ♂♀ CS (117)	FBG, HbA1c, meds	0.40 (0.09, 1.91)	NR
Tanaka et al. 2011	PCB180	Japan [Saku] SCOP, ♂♀ CS (117)	FBG, HbA1c, meds	1.76 (1.01, 3.08)	NR
Tanaka et al. 2011	PCB182/187	Japan [Saku] SCOP, ♂♀ CS (117)	FBG, HbA1c, meds	1.15 (0.36, 3.64)	NR
Lee et al. 2011	PCB105	Sweden (Uppsala) PIVUS, 70 years, ♂♀ CS (1,016)	FBG, meds	2.5 (1.3, 5)	Q5 vs. Q1 pg/ml
Lee et al. 2011	PCB118	Sweden (Uppsala) PIVUS, 70 years, ♂♀ CS (1,016)	FBG, meds	2.9 (1.4, 6)	Q5 vs. Q1 pg/ml
Lee et al. 2011	PCB138	Sweden (Uppsala) PIVUS, 70 years, ♂♀ CS (1,016)	FBG, meds	2.3 (1.2, 4.7)	Q5 vs. Q1 pg/ml
Lee et al. 2011	PCB153	Sweden (Uppsala) PIVUS, 70 years, ♂♀ CS (1,016)	FBG, meds	2 (1, 3.9)	Q5 vs. Q1 pg/ml
Lee et al. 2011	PCB156	Sweden (Uppsala) PIVUS, 70 years, ♂♀ CS (1,016)	FBG, meds	2 (1, 4.2)	Q5 vs. Q1 pg/ml
Lee et al. 2011	PCB157	Sweden (Uppsala) PIVUS, 70 years, ♂♀ CS (1,016)	FBG, meds	2.3 (1.1, 4.8)	Q5 vs. Q1 pg/ml
Lee et al. 2011	PCB170	Sweden (Uppsala) PIVUS, 70 years, ♂♀ CS (1,016)	FBG, meds	2.4 (1.1, 5.2)	Q5 vs. Q1 pg/ml
Lee et al. 2011	PCB180	Sweden (Uppsala) PIVUS, 70 years, ♂♀ CS (1,016)	FBG, meds	2.8 (1.3, 5.9)	Q6 vs. Q1 pg/ml
Lee et al. 2011	PCB189	Sweden (Uppsala) PIVUS, 70 years, ♂♀ CS (1,016)	FBG, meds	1.9 (1, 3.9)	Q5 vs. Q1 pg/ml
Lee et al. 2011	PCB194	Sweden (Uppsala) PIVUS, 70 years, ♂♀ CS (1,016)	FBG, meds	1.9 (0.9, 3.8)	Q5 vs. Q1 pg/ml
Lee et al. 2011	PCB206	Sweden (Uppsala) PIVUS, 70 years, ♂♀ CS (1,016)	FBG, meds	1.5 (0.8, 3.0)	Q5 vs. Q1 pg/ml
Lee et al. 2011	PCB209	Sweden (Uppsala) PIVUS, 70 years, ♂♀ CS (1,016)	FBG, meds	1.4 (0.7, 2.8)	Q5 vs. Q1 pg/ml
Lee et al. 2011	PCB74	Sweden (Uppsala) PIVUS, 70 years, ♂♀ CS (1,016)	FBG, meds	1.8 (0.9, 3.7)	Q5 vs. Q1 pg/ml
Lee et al. 2011	PCB99	Sweden (Uppsala) PIVUS, 70 years, ♂♀ CS (1,016)	FBG, meds	1.7 (0.9, 3.2)	Q5 vs. Q1 pg/ml
Lee et al. 2011	PCBs	Sweden (Uppsala) PIVUS, 70 years, ♂♀ CS (1,016)	FBG, meds	2.1 (1.1, 4.4)	Q5 vs. Q1 pg/ml
Lee et al. 2011	PCB105	Sweden (Uppsala) PIVUS, 70 years, ♂♀ Pros (1,016)	FBG, meds	8 (0.9, 68.2)	Q5 vs. Q1 pg/ml
Lee et al. 2011	PCB118	Sweden (Uppsala) PIVUS, 70 years, ♂♀ Pros (1,016)	FBG, meds	3.6 (0.7, 18.8)	Q5 vs. Q1 pg/ml
Lee et al. 2011	PCB138	Sweden (Uppsala) PIVUS, 70 years, ♂♀ Pros (1,016)	FBG, meds	3.2 (0.8, 13.2)	Q5 vs. Q1 pg/ml
Lee et al. 2011	PCB153	Sweden (Uppsala) PIVUS, 70 years, ♂♀ Pros (1,016)	FBG, meds	1.7 (0.5, 6.2)	Q5 vs. Q1 pg/ml
Lee et al. 2011	PCB156	Sweden (Uppsala) PIVUS, 70 years, ♂♀ Pros (1,016)	FBG, meds	2.6 (0.7, 10.3)	Q5 vs. Q1 pg/ml
Lee et al. 2011	PCB157	Sweden (Uppsala) PIVUS, 70 years, ♂♀ Pros (1,016)	FBG, meds	2.9 (0.8, 10.9)	Q5 vs. Q1 pg/ml
Lee et al. 2011	PCB170	Sweden (Uppsala) PIVUS, 70 years, ♂♀ Pros (1,016)	FBG, meds	3.6 (0.8, 16.8)	Q5 vs. Q1 pg/ml
Lee et al. 2011	PCB180	Sweden (Uppsala) PIVUS, 70 years, ♂♀ Pros (1,016)	FBG, meds	4.6 (1.1, 18.8)	Q4 vs. Q1 pg/ml
Lee et al. 2011	PCB189	Sweden (Uppsala) PIVUS, 70 years, ♂♀ Pros (1,016)	FBG, meds	2.8 (0.7, 11.2)	Q5 vs. Q1 pg/ml
Lee et al. 2011	PCB194	Sweden (Uppsala) PIVUS, 70 years, ♂♀ Pros (1,016)	FBG, meds	6 (1.6, 22.9)	Q5 vs. Q1 pg/ml
Lee et al. 2011	PCB206	Sweden (Uppsala) PIVUS, 70 years, ♂♀ Pros (1,016)	FBG, meds	5.5 (1.4, 21.9)	Q4 vs. Q1 pg/ml
Lee et al. 2011	PCB209	Sweden (Uppsala) PIVUS, 70 years, ♂♀ Pros (1,016)	FBG, meds	6.2 (1.2, 33)	Q5 vs. Q1 pg/ml
Lee et al. 2011	PCB74	Sweden (Uppsala) PIVUS, 70 years, ♂♀ Pros (1,016)	FBG, meds	8.5 (1.0, 71.8)	Q3 vs. Q1 pg/ml
Lee et al. 2011	PCB99	Sweden (Uppsala) PIVUS, 70 years, ♂♀ Pros (1,016)	FBG, meds	3.3 (0.6, 17.3)	Q5 vs. Q1 pg/ml
Lee et al. 2011	PCBs	Sweden (Uppsala) PIVUS, 70 years, ♂♀ Pros (1,016)	FBG, meds	5.1 (1.0, 26)	Q3 vs. Q1 pg/ml
Persky et al. 2011	PCB, anti-estrog.	USA (LaSalle County, Illinois) capacitor plant, ♀ CS (118)	Self-report	12.3, p=0.0042 (OR, p-value)	previous workers vs. local residents
Persky et al. 2011	PCB, estrog.	USA (LaSalle County, Illinois) capacitor plant, ♀ CS (118)	Self-report	3.4, p=0.0120 (OR, p-value)	previous workers vs. local residents
Persky et al. 2011	PCBs	USA (LaSalle County, Illinois) capacitor plant, ♀ CS (118)	Self-report	4.4 , p=0.0185 (OR, p-value)	previous workers vs. local residents
Persky et al. 2011	PCBs	USA (LaSalle County, Illinois) capacitor plant, ♀ CS (118)	Self-report	4.4 , p=0.0184 (OR, p-value)	previous workers vs. local residents
Persky et al. 2011	PCBs, dioxin-like	USA (LaSalle County, Illinois) capacitor plant, ♀ CS (118)	Self-report	10, p=0.0035 (OR, p-value)	previous workers vs. local residents
Persky et al. 2011	PCBs, non-dioxin	USA (LaSalle County, Illinois) capacitor plant, ♀ CS (118)	Self-report	4, p=0.0251 (OR, p-value)	previous workers vs. local residents

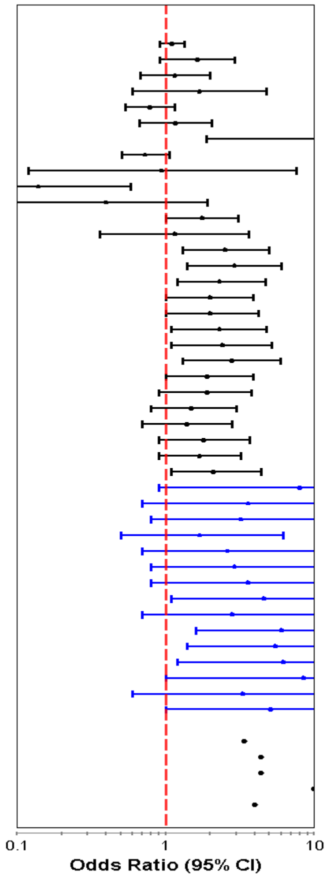

cross-sectional   prospective

Abbreviations: PIVUS, Prospective Investigation of the Vasculature in Uppsala Seniors study; SCOP, Saku Control Obesity Program; Pros – prospective or nested case control; CS, cross-sectional; OR, odds ratio; FBG, Fasting blood glucose; meds, medications used to treat type 2 diabetes; OGTT, glucose tolerance test; HbA1c, Glycated haemoglobin; FBG, HbA1c, 2hr glucose, levels are sufficiently elevated to be classified as type 2 diabetes; %ile, percentile; Q, quantile; NR, not reported. [a]Values are adjusted ORs unless otherwise noted. [b]If no lipid adjustments were reported, the OR was not lipid adjusted; exposures were measured in serum samples unless otherwise indicated.

References Cited

Airaksinen R, Rantakokko P, Eriksson JG, Blomstedt P, Kajantie E, Kiviranta H. 2011. Association Between Type 2 Diabetes and Exposure to Persistent Organic Pollutants. Diabetes Care 34(9):1972-1979.

Codru N, Schymura MJ, Negoita S, Rej R, Carpenter DO. 2007. Diabetes in relation to serum levels of polychlorinated biphenyls and chlorinated pesticides in adult Native Americans. Environ Health Perspect 115(10):1442-1447.

Everett CJ, Frithsen IL, Diaz VA, Koopman RJ, Simpson WM, Jr., Mainous AG, 3rd. 2007. Association of a polychlorinated dibenzo-p-dioxin, a polychlorinated biphenyl, and DDT with diabetes in the 1999-2002 National Health and Nutrition Examination Survey. Environ Res 103(3):413-418.

Grandjean P, Henriksen JE, Choi AL, Petersen MS, Dalgard C, Nielsen F, et al. 2011. Marine food pollutants as a risk factor for hypoinsulinemia and type 2 diabetes. Epidemiology 22(3):410-417.

Lee D-H, Steffes MW, Sjodin A, Jones RS, Needham LL, Jacobs JDR. 2010. Low dose of some persistent organic pollutants predicts type 2 diabetes: A nested case-control study. Environ Health Perspect 118(9):1235-1242.

Lee DH, Lind PM, Jacobs DR, Jr., Salihovic S, van Bavel B, Lind L. 2011. Polychlorinated biphenyls and organochlorine pesticides in plasma predict development of type 2 diabetes in the elderly: The Prospective Investigation of the vasculature in Uppsala Seniors (PIVUS) study. Diabetes Care 34(8):1778-1784.

Patel CJ, Bhattacharya J, Butte AJ. 2010. An environment-wide association study (EWAS) on type 2 diabetes mellitus. PLoS ONE 5(5):e10746.

Persky V, Piorkowski J, Turyk M, Freels S, Chatterton Jr R, Dimos J, et al. 2011. Associations of polychlorinated biphenyl exposure and endogenous hormones with diabetes in post-menopausal women previously employed at a capacitor manufacturing plant. Environmental Research 111(6):817-824.

Philibert, A., H. Schwartz, et al. (2009). "An exploratory study of diabetes in a First Nation community with respect to serum concentrations of p,p'-DDE and PCBs and fish consumption." International journal of environmental research and public health **6**(12): 3179-3189.

Turyk M, Anderson H, Knobeloch L, Imm P, Persky V. 2009. Organochlorine exposure and incidence of diabetes in a cohort of Great Lakes sport fish consumers. Environ Health Perspect 117(7):1076-1082.
